# Transcriptome sequencing revealed the influence of blue light on the expression levels of light-stress response genes in *Centella asiatica*

**DOI:** 10.1371/journal.pone.0260468

**Published:** 2021-11-29

**Authors:** Wanapinun Nawae, Thippawan Yoocha, Nattapol Narong, Atchara Paemanee, Yanisa Ketngamkum, Kanokwan Romyanon, Theerayut Toojinda, Sithichoke Tangphatsornruang, Wirulda Pootakham

**Affiliations:** 1 National Omics Center (NOC), National Science and Technology Development Agency, Thailand Science Park, Pathum Thani, Thailand; 2 National Center for Genetic Engineering and Biotechnology (BIOTEC), National Science and Technology Development Agency, Thailand Science Park, Pathum Thani, Thailand; CSIR-Institute of Himalayan Bioresource Technology: Institute of Himalayan Bioresource Technology CSIR, INDIA

## Abstract

*Centella asiatica* is rich in medical and cosmetic properties. While physiological responses of *C*. *asiatica* to light have been widely reported, the knowledge of the effects of light on its gene expression is sparse. In this study, we used RNA sequencing (RNA-seq) to investigate the expression of the *C*. *asiatica* genes in response to monochromatic red and blue light. Most of the differentially expressed genes (DEGs) under blue light were up-regulated but those under red light were down-regulated. The DEGs encoded for CRY-DASH and UVR3 were among up-regulated genes that play significant roles in responses under blue light. The DEGs involved in the response to photosystem II photodamages and in the biosynthesis of photoprotective xanthophylls were also up-regulated. The expression of flavonoid biosynthetic DEGs under blue light was up-regulated but that under red light was down-regulated. Correspondingly, total flavonoid content under blue light was higher than that under red light. The *ABI5*, *MYB4*, and *HYH* transcription factors appeared as hub nodes in the protein-protein interaction network of the DEGs under blue light while *ERF38* was a hub node among the DEGs under red light. In summary, stress-responsive genes were predominantly up-regulated under blue light to respond to stresses that could be induced under high energy light. The information obtained from this study can be useful to better understand the responses of *C*. *asiatica* to different light qualities.

## Introduction

*Centella asiatica* (L.) Urban is a plant in the family Apiaceae [1]. It shows several biological activities that are pharmaceutically and cosmetically useful [2, 3]. The extracts from this plant have been reported to have wound healing [4], antioxidant [5], anti-inflammatory [6], antibacterial [7], anticancer [8], and neuroprotective [9] activities. *C*. *asiatica* is also a rich source of saponins, triterpenes, phytosterols, caffeoylquinic acids, and flavonoids, which are beneficial to human health [10].

Light can significantly affect plant morphology and metabolism [11]. Many studies have been conducted to understand the effect of light quality on plant growth and the production of several secondary metabolites [12, 13]. In *C*. *asiatica*, UV-B radiation was shown to increase leaf yield [14]. High light intensity was reported to increase the concentrations of flavonoids, anthocyanins, and saponins in *C*. *asiatica* [14]. In contrast, low light intensity was shown to reduce asiaticoside and madecassoside contents of three *C*. *asiatica* accessions from Thailand [15]. The *C*. *asiatica* offspring ramets that were treated with low light showed increased biomass and stolon length [16].

While the effect of light on biomass and secondary metabolite productions are well studied, little is known about the effect of light quality on the expression of *C*. *asiatica* genes. RNA sequencing (RNA-Seq) method has been used to examine gene expression of tea plants in response to blue, purple, and yellow light treatments [17]. In lettuce, the analysis of RNA-Seq data showed the downregulation of genes involved in flavonoid biosynthesis under green light [18]. A recent report on the *C*. *asiatica* genome [19] provides a reference sequence and gene annotation that enables the genome-wide gene expression patterns to be precisely analyzed using RNA-Seq data.

In this study, we used the RNA-Seq method to investigate the expression of *C*. *asiatica* genes in response to monochromatic red or blue light. As a result, we found several differentially expressed genes (DEGs) that were implicated in several biological pathways. Many of these genes were regulated in the opposite direction under red and blue light.

## Materials and methods

### Plant materials

Whole plants of *C*. *asiatica* derived from node segments of stolons were hydroponically grown under a controlled environment in Enshi medium solution [20], with EC 1.8 mS cm^-1^, pH 5.0–6.0 at 26 ± 2°C, the relative humidity ranged from 55% to 60%. Composition of Enshi medium solution was shown in S1 Table. They were cultured under a white light from light emitting diode (LED) at an intensity of 150 mmol m^–2^ s^–1^ during the 12-hour photoperiod for 120 days and were used for all treatments. LED light sources were used to provide different light conditions. In the control treatment (white light ~150 μmol m^−2^ s ^−1^, 580–680 nm), whole plant materials were grown under 12-hour light/dark cycles. Red light samples (~60 μmol m^−2^ s ^−1^, 580–680 nm) and blue light samples (~60 μmol m^−2^ s ^−1^, 400–480 nm) were continuously exposed to their respective light conditions, and the materials used for RNA preparation were collected after 5 days (S1 Fig). For each of the white, red, and blue light conditions, *C*. *asiatica* leaf samples were collected from three plants for replication.

### RNA extraction, cDNA library construction and sequencing

The *C*. *asiatica* leaf samples were pulverized in liquid nitrogen. For each sample, total RNA was extracted with the CTAB method. DNA-*free*^™^ DNA Removal Kit (Invitrogen^™^) was used to remove contaminated DNA. The quality and quantity of RNA was evaluated with the fragment analyzer machine (Agilent). Dynabeads^®^ mRNA Purification Kit (Invitrogen^™^) was used to purify mRNA. We constructed cDNA libraries according to the MGIEasy RNA Library Prep set protocol. The libraries were sequenced with the MGISEQ-2000RS machine.

### RNA read mapping and differential gene expression analysis

We mapped the RNA reads to the *C*. *asiatica* reference genome with HISAT2 [21]. The reference genome sequence was downloaded from the national center for biotechnology information (NCBI) database with the BioProject number PRJNA642665 [19]. StringTie2 was used to quantify RNA reads mapped to gene regions of the genome [22]. The gene expression levels were compared with DESeq2 to identify DEGs [23]. The gene expression levels under monochromatic red and blue light were compared with those under white light. The gene expression level was also compared between *C*. *asiatica* under red and blue light. As a result, three sets of DEGs were obtained. The DEGs with log2 fold-change greater than 1.5 at the adjusted *p*-value cutoff of 0.05 were retained for subsequent analyses. We used three biological replicates in all DESeq2 analyses.

### Analysis of the differentially expressed gene function

We used BLAST software to find the homologous functions of the DEG encoded proteins based on the NCBI and the universal protein resource (UniProt) databases. The gene ontology (GO) terms of the DEGs were extracted from the blast results. We used Mercator4 (or MapMan4) to classify the DEGs into functional classes [24]. The GO enrichment was analyzed with AgriGO 2.0 [25]. The pathways in which the DEG encoding proteins were involved were identified and visualized based on data from the Kyoto encyclopedia of genes and genomes (KEGG) database [26].

### Gene regulatory network analysis

The regulatory interaction among the DEGs was obtained from the plant transcriptional regulatory map (PlantRegMap) database [27] based on the sequence similarity search with the protein sequences of *Arabidopsis thaliana* and *Daucus carota*. We also searched for the homologs of the DEGs from the search tool for the retrieval of interacting genes/proteins (STRING) database [28]. The protein-protein interaction networks were obtained mainly based on the co-expression information from the database. The data from the PlantRegMap and STRING databases were combined. The networks were visualized and analyzed with Cytoscape software [29].

### Total flavonoids measurement

For each of nine *C*. *asiatica* samples, the extract was diluted with 96% ethyl alcohol in a 1: 3 ratio and then mix with 10 μl of 10% aluminum chloride solution. The solution of each sample was then added with 150 μl of 96% ethyl alcohol and 10 μl of one molar sodium acetate and was incubated in the dark at room temperature for 40 minutes. The absorbance of standard solutions with quercetin at the concentration of 10, 20, 40, 60, 80, 100, 120 μg/ml was measured at 415 nm. The absorbance of each sample was then compared with the absorbance curve of such standard quercetin solutions. Thereby, total flavonoid content in each sample was expressed as milligram quercetin equivalent per gram of sample dry weight (mg QE/g) [30]. The difference in flavonoid content between *C*. *asiatica* groups was analyzed with the one-tailed t-test and was visualized with the ggpubr package of R language.

### Gene expression analysis by RT-qPCR

We selected ten DEGs, including Cryptochrome DASH (*CaCRYD*), UV repair defective 3 (*CaUVR3*), Blue-light inhibitor of cryptochromes 1 (*CaBIC1*), Early light-induced protein 1 (*CaELIP1*), Violaxanthin de-epoxidase (*CaVDE*), ABA DEFICIENT 4 (*CaABA4*), Phenylalanine ammonia-lyase 1 (*CaPAL1*), Chalcone—flavanone isomerase 3 (*CaCHIL*), Flavanone 3-hydroxylase (*CaF3H*), and ABSCISIC ACID-INSENSITIVE 5 (*CaABI5*), for reverse transcription-quantitative polymerase chain reaction (RT-qPCR) analysis. Glyceraldehyde 3-phosphate dehydrogenase (*GAPDH*) was used as a reference housekeeping gene. Primers were designed with the PrimerQuest Tool (S2 Table). For RT-qPCR analysis, 50 ng of RNA was used as a template in a 20 μL total reaction. The cycling condition was set according to the EvoScript RNA SYBR^®^ Green I Master (Roche, Germany) instructions. The reactions were done in QuantStudio^™^ 6 Flex Real-Time PCR System. All samples were run with three biological replicates, and three technical replicates for each PCR reaction. The 2^-ΔΔCt^ method was used to calculate gene expression changes [31]. The unpaired Student’s *t*-test at p-value cutoff of 0.05 was conducted with the ggpubr package. Scatter plot of the expression values from RNA-seq and RT-qPCR data was done with Microsoft Excel.

## Results and discussion

### Different effects of red and blue light on *C*. *asiatica* gene expressions

**Fig 1:**
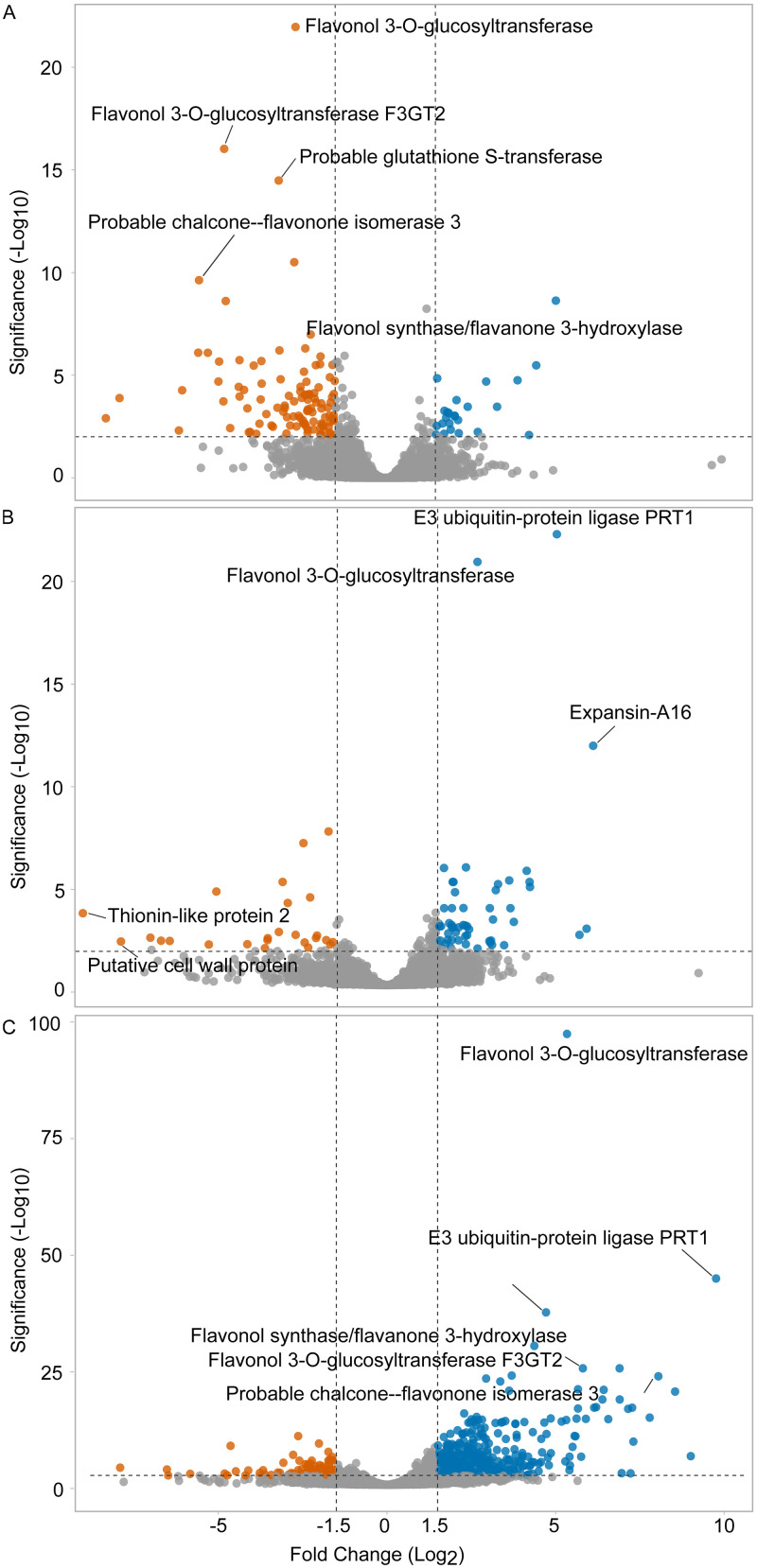
Volcano plot of RNA-Seq data. Scatter plots show the log2 of fold change and significant levels of genes from the expression comparison between (A) the R-treated and the W-treated *C*. *asiatica*, (B) the B-treated and the W-treated *C*. *asiatica*, and (C) the B-treated and the R-treated *C*. *asiatica*. The grey dots show insignificant DEGs (|log2 of fold change| < 1.5 and/or FDR > 0.05). Blue dots show genes of the R-treated or the B-treated *C*. *asiatica* with a higher expression level than that of the references (the W-treated in panel A and B, and the B-treated in panel C). Orange dots show genes of the R-treated or the B-treated *C*. *asiatica* with a lower expression level than that of the references.

We sequenced RNA from the *C*. *asiatica* treated with red (R-treated), blue (B-treated), or white (W-treated) light. The sequenced reads were mapped to the *C*. *asiatica* reference sequence [19]. We were able to map 95–96% of the total RNA reads to the reference genome (S3 Table). The mapped RNA reads covered up to 80% (22,228 genes) of the total annotated genes of the reference genome. The number of mapped reads per gene represented the expression level of each gene. The gene expression levels under red and blue light were compared with those under white light (control condition). A gene whose expression level under red or blue light was higher than that under white light was referred to as an up-regulated DEG, and *vice versa* for a down-regulated DEG (Fig 1A and 1B). Under red light, the number of down-regulated DEGs (red-downDEGs) was higher than the number of the up-regulated DEGs (red-upDEGs) (S2A Fig). In contrast, the number of down-regulated DEGs under blue light (blue-downDEGs) was lower than the number of the up-regulated DEGs (blue-upDEGs). The expression levels were also compared between the R-treated and the B-treated *C*. *asiatica* (Fig 1C). The R-treated *C*. *asiatica* showed 205 genes with a higher expression level than that of the B-treated ones (red-hiDEGs) (S2B Fig). Other 618 genes were expressed at higher levels under blue light than that under red light (blue-hiDEGs) (S2C Fig). These results revealed that a majority of *C*. *asiatica* genes were down-regulated under red light but were up-regulated under blue light. Similar gene expression profile has been reported in Norway spruce [32].

**Fig 2:**
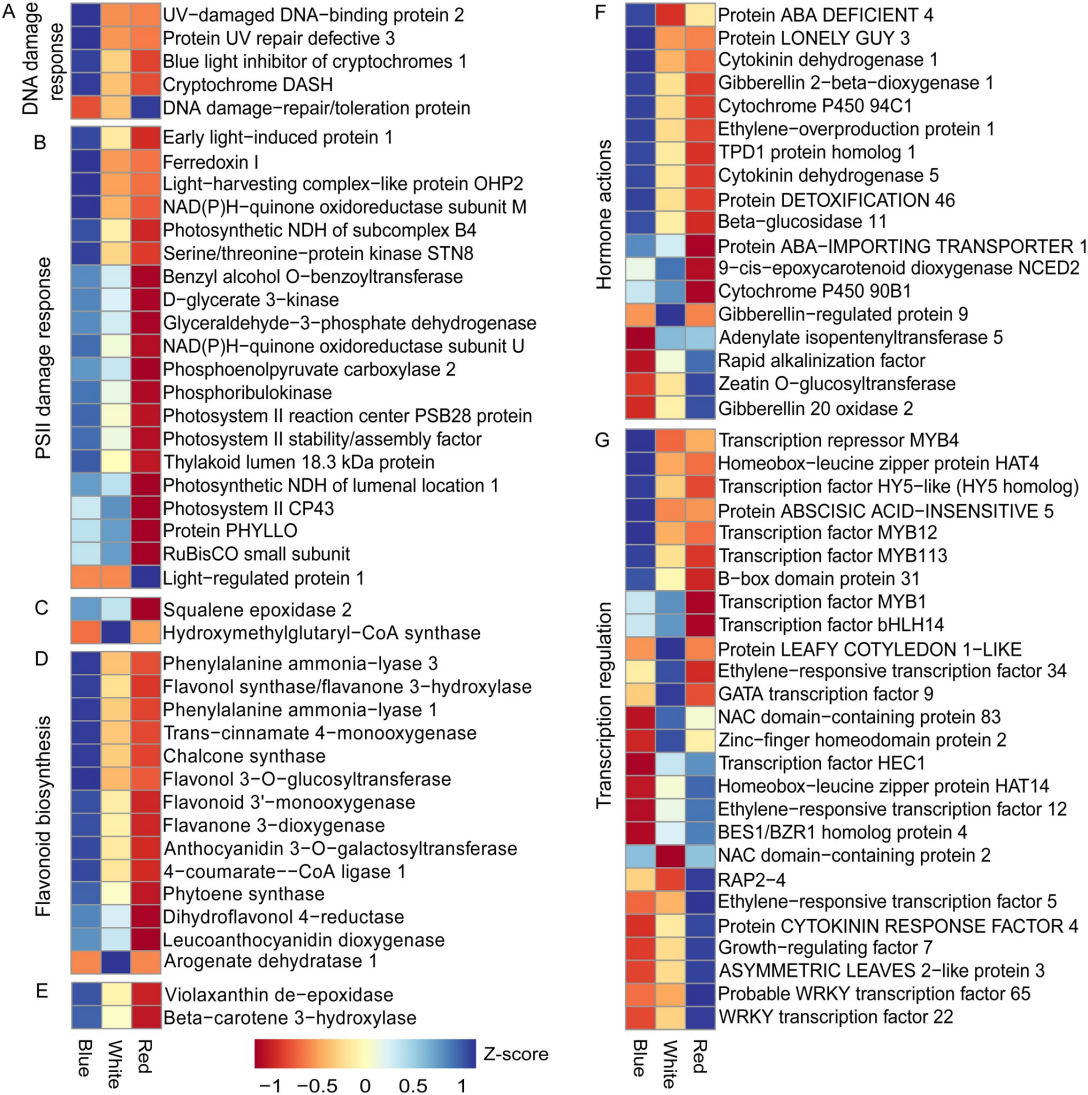
The expression levels of the functionally correlated DEGs. The DEGs are involved in (A) the responses to the DNA damages, (B) the responses to the PSII damages, (C) mevalonic acid pathway, (D) flavonoid biosynthesis, (E) carotenoid biosynthesis, (F) hormone actions, and (G) transcription regulation. The expression level of each DEG is shown as the Z-score of its transcripts per kilobase million (TPM) value. The Z-scores are calculated in each row to scale the expression level of each DEG based light conditions from high (blue color) to low (brown color).

We analyzed overrepresented gene ontology (GO) terms and classified the DEGs into four functional groups (Table 1). A majority of the DEGs within these four classified groups were down-regulated in the R-treated *C*. *asiatica* and were up-regulated in the B-treated plants, according to the number of up- and down-regulated DEGs under each light condition (Fig 2 and S4 Table).

**Table 1:** The overrepresented GO terms of each DEG set.

	GO	Description	red-downDEGs	blue-upDEGs	red-hiDEGs	blue-hiDEGs
**Group 1**	GO:0009637	response to blue light	-	-	-	0.022
	GO:0009642	response to light intensity	-	0.0015	-	0.00023
	GO:0071482	cellular response to light stimulus	-	8.30E-06	-	-
	GO:0009785	blue light signaling pathway	-	-	-	0.00035
	GO:0009411	response to UV	-	-	-	0.04
**Group 2**	GO:0009699	phenylpropanoid biosynthetic process	-	0.000098	-	0.0014
	GO:0009813	flavonoid biosynthetic process	0.00005	-	-	0.0019
	GO:0006558	L-phenylalanine metabolic process	-	-	-	0.008
	GO:0016209	antioxidant activity	0.042	-	-	0.04
	GO:0098869	cellular oxidant detoxification	0.044	-	-	0.043
**Group 3**	GO:0009772	photosynthetic electron transport in photosystem II	-	-	-	1.10E-17
	GO:0015979	photosynthesis	0.0097	-	-	7.3E-20
	GO:0022900	electron transport chain	-	-	-	7.40E-10
	GO:0042548	regulation of photosynthesis, light reaction	-	-	-	0.0002
	GO:0009055	electron carrier activity	-	-	-	1.30E-09
**Group 4**	GO:0009755	hormone-mediated signaling pathway	-	-	0.03	-
	GO:0003700	transcription factor activity, sequence-specific DNA binding	-	-	0.0097	-
	GO:0009690	cytokinin metabolic process	-	-	-	0.00023

The p-values of the GO enrichment analysis in each of red-downDEG, blue-upDEG, red-hiDEG, and blue-hiDEG sets are shown. The lower p-value represent a higher significance of the enrichment of that GO term. Dash symbol shows the insignificance of a GO term.

For the DEGs in Group 1 (Table 1), the enriched GO terms suggested the impact of high-energy light on gene expression under blue light. In this group, we found the up-regulation of Early light-induced protein 1 (*CaELIP1*) gene in the B-treated *C*. *asiatic* (Fig 2 and S4 Table). This gene was down-regulated in the R-treated *C*. *asiatic*. The *ELIP* expression has been shown in other plants to be induced by blue light (but not by red or far-red light) and high light energy [33, 34]. *ELIP1* could protect plants from photooxidative stress [35]. Transcription factors (TF), such as ELONGATED HYPOCOTYL 5 homolog (*HY5* homolog or *CaHYH*) and B-box domain protein 31 (*CaBBX31*), had a similar expression profile to *CaELIP1* (Fig 2B and S4 Table). These TFs have been reported to regulated *ELIP1* expression [36–38]. The expression of *BBX31* was also shown to depend on light energy [38]. For Group 2 (Table 1), several flavonoid biosynthetic DEGs were up-regulated in the B-treated *C*. *asiatic* but were down-regulated in the R-treated plants. Similar expression profile has been reported in other plant [32]. A high-light protection function of flavonoids might contribute to the high expression of these genes under blue light, which had a higher energy level than red light [39]. The production of flavonoids under stress, however, could compete with primary metabolisms for carbon and energy sources [40, 41]. The enriched GO terms of the DEGs in Group 3 indicated that the expression of the genes that were involved in the photosynthesis and electron transport under blue light was higher than that under red light (Table 1). It has been shown that CO_2_ assimilation, photosystem II (PSII) electron transportation, and photosynthesis in plants treated with red light were impaired to a greater extent than the plants treated with blue light [42, 43]. Ribose-1, 5-bisphosphate carboxylase/oxygenase (Rubisco) activity under red light was also lower than that under blue light [43]. In this study, the expression of gene encoding Rubisco small subunit under blue light was higher than that red light (Fig 2B and S4 Table). The down-regulation of flavonoid biosynthetic genes under red light might be correlated with the decreased expression of photosynthetic genes, which could negatively affect the photosynthetic efficiency of the R-treated *C*. *asiatic*. For Group 4, the DEGs were involved in hormone metabolism (Table 1). Gibberellin 2-beta-dioxygenase 1 (*CaGA2OX1*) gene was down-regulated under red light but were up-regulated under blue light (Fig 2F and S4 Table). The expression of *CaGA2OX2* and *CaGA2OX8* under blue light was also higher than that under red light (S4 Table). The transcription of *GA2OX2* could be reduced with red light treatment [44]. The expression of *GA2OX8* was up-regulated with high light [45]. This gene could inactivate gibberellins (GA) and affect plant growth and development under high light [46].

Overall, the stresses and signal that could be induced under high-energy light might contribute to the up-regulation of several TF, signaling, stress responsive genes in the B-treated *C*. *asiatic*. In contrast, the down-regulation of stress responsive genes in the R-treated *C*. *asiatic* suggested a lower level of such stresses under low-energy light and the reduced expression of genes involved in secondary metabolite production might reduce the consumption of carbon and energy under photosynthetic inefficient status.

### Gene expression of the photoreceptor genes under blue and red light

The expression of photoreceptor phytochrome A (*PHYA*), phototropin1, 2 (*PHOT1*,*2*), and cryptochrome 2 (*CRY2*) encoding genes was not significantly different among the *C*. *asiatica* treated with white, red, or blue light for five days. Similar results have been found in other studies and it has been suggested that the expression of photoreceptor genes might not be specific to particular light spectrums [32, 47]. For example, the expression of *PHOT1* (blue light-sensitive photoreceptor) under blue light was lower than that under white or red light, while *PHOT2* was expressed at a similar level under red and blue light [48]. The expression of *PHYA* (red light-sensitive photoreceptor) could decrease under red light treatments [49, 50]. Phytochrome B was shown to be able to receive blue light in some circumstance [51]. In this study, the expression of phytochrome B (*CaPHYB*) encoding genes in the B-treated *C*. *asiatica* was higher than that in the R-treated plants (S4 Table).

Although the expression of those photoreceptor genes was insignificantly changed, the expression of genes encoded for proteins whose functions related to photoreceptor activity was significant altered. We found an up- and down-regulation of Blue‐light inhibitor of cryptochromes 1 (*CaBIC1*) encoding gene in the B- and the R-treated *C*. *asiatica*, respectively (Fig 2A). In Arabidopsis, the expression of *BIC1* under blue light was also higher than that under red light [52]. Photoactivation of Cryptochrome 2 (CRY2) activated the transcription of *BIC* genes, which in turn suppressed photoactivated CRY2 [52–54]. Plants have a negative feedback mechanism to regulate the activity of proteins in light signaling pathway [52, 55]. The insignificant change of *CaCRY2* expression in the B-treated *C*. *asiatica* might be related to the up-regulation of *CaBIC1*, which could negatively regulated role on this blue receptor [52–54]. The negative feedback mechanism might also play a role on the expression of other photoreceptors of the B- and the R-treated *C*. *asiatica*.

### The upregulation of genes involved in the protection of DNA and PSII photodamages under blue light

We found several DEGs with the functions involved in the repair of light-induced DNA damages (Fig 2A and S4 Table). The long exposure to blue light was related to the occurrence of cyclobutane pyrimidine dimers (CPDs) and pyrimidine-pyrimidone 6–4 photoproducts (6–4 PPs) DNA damages in both nuclear and organellar genomes of Arabidopsis leaves [56]. In this study, Cryptochrome DASH (*CaCRYD* or *CaCRY3*) encoding gene was up-regulated under blue light but was down-regulated under red light (Fig 2A and S4 Table). The protein domain analysis showed that *CaCRY3* protein contained FAD-binding domain in its N-terminal photolyase-homologous region (PHR), which was essential for blue light absorption, but lacked C-terminal extension region (CCE), which was required for light signal transduction [57]. Another up-regulated gene under blue light encoded for protein UV repair defective 3 (*CaUVR3*), which has similar domain architecture as CaCRY3. CRY3 and UVR3 as (6–4)DNA photolyase proteins exhibited light-driven DNA repair [58]. CRY3 was reported to correct CPDs [58], while (6–4)DNA photolyase protein repaired 6–4 PPs damages [59]. We also found the upregulation of the UV-damaged DNA-binding protein 2 (*CaDDB2*) gene under blue light. DDB2, in complex with DDB1, was reported to recognize CPD lesions in human cell [60].

Several other DEGs were involved in the response to PSII photodamages (Fig 2B and S4 Table). These DEGs were up-regulated under blue light but were down-regulated under red light. The gene encoded for State transition 8 protein (*CaSTN8*) was one of these DEGs (Fig 2B and S4 Table). STN8 specifically phosphorylated photosystem II protein psbD/D2, psbC/CP43 and other core proteins of PSII [61]. Correspondingly, we found a higher expression of *psbD* and *psbC* under blue light than that under red light. The phosphorylation mediated by STN8 was essential for PSII repair [62, 63]. Early light-induced protein 1 encoding gene (*CaELIP1*) was also a member of this group [33]. The accumulation of *ELIP1* products was shown to be correlated with the level of light energy absorbed by plants and with the degree of photoinactivation and photodamage of PSII [33, 34]. The expression of *ELIP1* was shown to be regulated by the signaling pathway, in which the photoreceptor UV-B resistance 8 (UVR8) was involved [36]. UVR8 could absorb blue light photons and induced gene expression under blue light [64]. Correspondingly, we found the upregulation of genes encoded for *CaUVR8* in the B-treated *C*. *asiatica* (Fig 2A and S4 Table).

**Fig 3:**
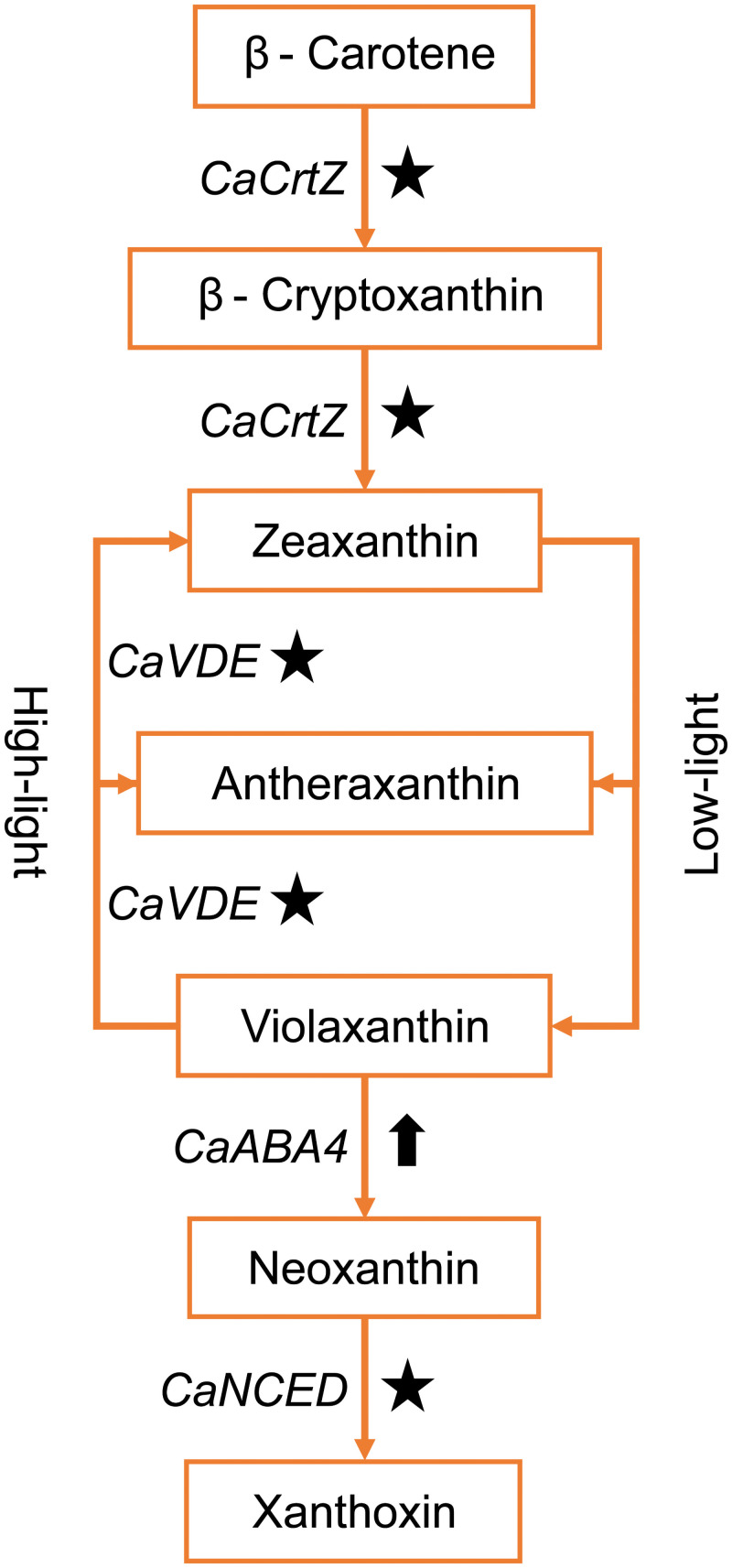
The DEGs involved in the biosynthesis of xanthophylls under blue light. The sub-pathway involved in the biosynthesis of zeaxanthin, neoxanthin, and xanthoxin is obtained from the carotenoid biosynthesis pathway of the KEGG database. The DEG implicated in each step is shown. Star symbol indicates that the expression level under blue light of the corresponding DEG is higher than that under red light. Up-arrow symbol shows that the corresponding DEG is up-regulated under blue light. *CaCrtZ*: beta-carotene hydroxylase, *CaVDE*: violaxanthin de-epoxidase, *CaABA4*: abscisic acid-deficient 4, *CaNCED*: 9-cis-epoxycarotenoid dioxygenase.

The DEGs of the carotenoid biosynthesis pathway were also expressed at higher levels in the B-treated *C*. *asiatica* than those in the R-treated ones (Figs 2E and 3). Carotenoids could function as photoprotectors and facilitators for the assembly of photosystems and light harvesting antenna complexes [65]. The expression of Beta-carotene hydroxylase encoding gene (*CaCrtZ*) under blue light was higher than that under red light condition (Fig 3). Under blue light, we found the up-regulated DEGs encoded for violaxanthin de-epoxidase (*CaVDE* or *CaNPQ1*), which was involved in the conversion of violaxanthin to zeaxanthin under high-light condition (Fig 3) [66]. This gene could alleviate photoinhibition and lipid peroxidation under excess light because zeaxanthin could quench singlet excited chlorophyll, reduce triplet excited chlorophyll formation, and scavenge reactive oxygen species [67, 68]. We also found the upregulation of the Abscisic acid-deficient 4 encoding gene (*CaABA4*) under blue light (Fig 3). *ABA4* played a role in the biosynthesis of neoxanthin [69]. Similarly, 9-cis-epoxycarotenoid dioxygenase encoding gene (*CaNCED*), which was highly expressed under blue light (Fig 3). This enzyme was responsible for the biosynthesis of xanthoxin [70], which played a role in the photoprotection of PSII [71]. Together, these results suggest that continuous exposure to blue light might cause DNA and PSII photodamages in the B-treated *C*. *asiatica* and induced the expression of related stress-responsive genes. The down-regulation of those stress-responsive genes in the R-treated *C*. *asiatica* suggested that the level of DNA and PSII photodamages under red light might be lower than that under blue or white light, which contained blue spectrum.

### The expression of flavonoid biosynthetic genes and their key transcription factors

**Fig 4:**
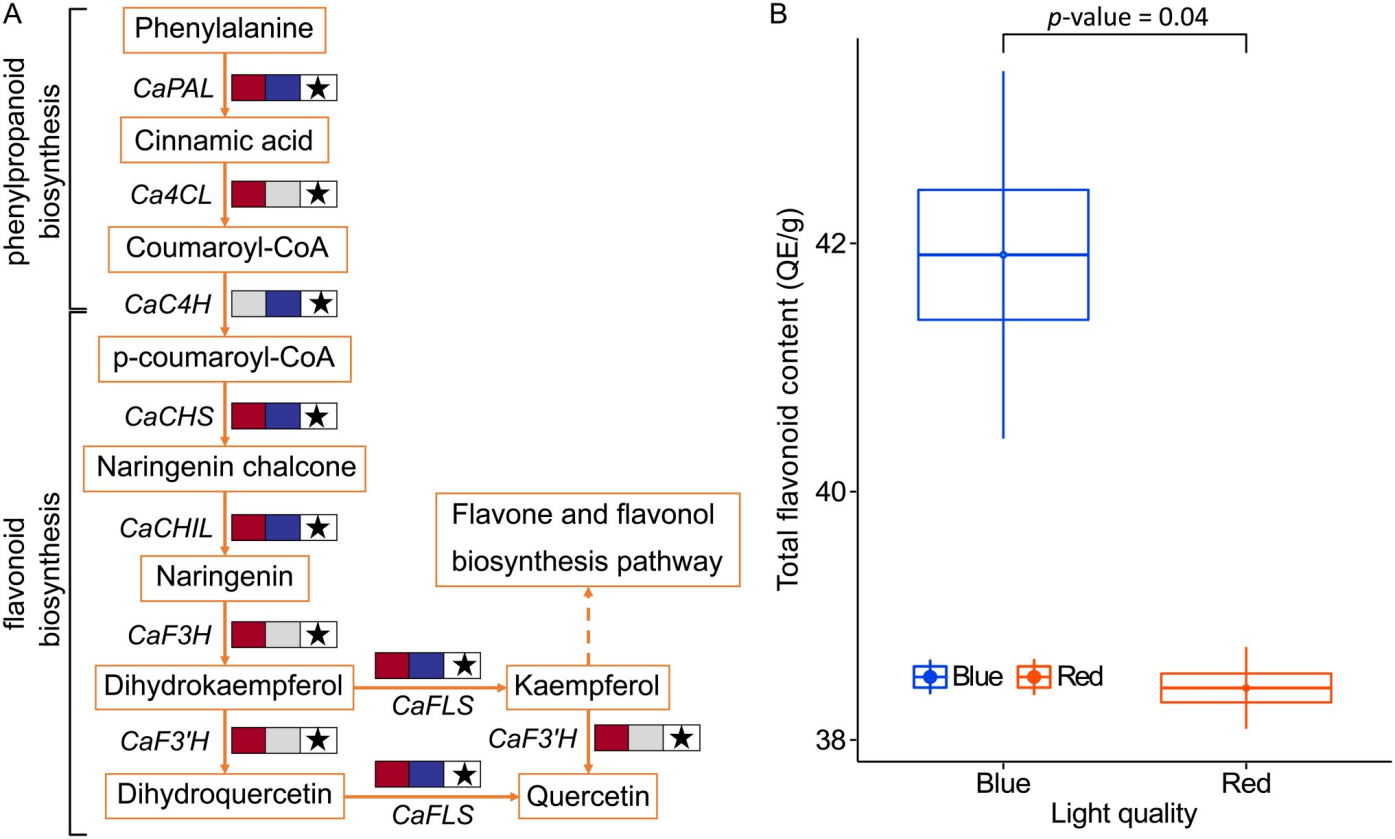
The DEGs involved in flavonoid biosynthesis. (A) The sub-pathway involved in the flavonoid biosynthesis is obtained from the phenylpropanoid biosynthesis pathway and the flavonoid biosynthesis pathway of the KEGG database. Dashed box covers flavonoid biosynthesis pathway. The enzyme encoded by each DEG implicated in each step is shown. The first of the colored boxes shows the expression change of the DEG from the expression comparison between the R-treated and the W- treated *C*. *asiatica*. The second box shows the expression change of the DEG from the expression comparison between the B-treated and the W- treated *C*. *asiatica*. Up- and down-regulated DEGs are shown in blue and brown, respectively. Light grey shows that the corresponding DEG is insignificantly changed under a particular condition. Star symbol indicates that the expression level under blue light of the corresponding DEG is higher than that under red light. (B) Total flavonoid contents of *C*. *asiatica* leaves under white, blue, and red light are shown. Significant difference of flavonoid contents between light conditions is shown with *p*-value. *CaPAL*: Phenylalanine ammonia-lyase 1, *Ca4CL*: 4-coumarate—CoA ligase 1, *CaC4H*: cinnamate 4-hydroxylase, *CaCHS*: cinnamate 4-hydroxylase, *CaCHIL*: chalcone-flavanone isomerase 3, *CaF3H*: Flavanone 3-hydroxylase, *CaF3’H*: Flavonoid 3’-hydroxylase, *CaFLS*: flavonol synthase.

The expression of genes involved in the flavonoid biosynthesis pathway was notably different between the R-treated and the B-treated *C*. *asiatica* (Table 1 and Fig 4A). Under red light, the expression of all DEGs of the flavonoid biosynthesis pathway was down-regulated and was lower than that under blue light (Figs 2D and 4A). The expression of several of these genes, for example, Flavanone 3-hydroxylase (*F3H*) and Flavonoid 3’-hydroxylase (*F3’H*) was shown to be strongly correlated with the level of flavonoid content [72]. In wheat sprout, the levels of *F3’H* and Phenylalanine ammonia-lyase 1 (*PAL1*) expressions and flavonoid content under blue was higher than those under red light [73]. Accordingly, we found in this study that the level of total flavonoid content under red light (38.4±0.3 mg QE/g) was significantly lower than that under blue light (41.9±1.5 mg QE/g) (Fig 4B). In pea, flavonoid contents were also shown to be highly responsive to blue light [74]. Plant flavonols, such as quercetin had an ability to absorb short wavelength light and could protect DNA molecules from high energy light [75]. Flavonoids in terrestrial plants were proposed to function as antioxidants, signaling transmitters, phytohormone action regulators, and high light protectors [39]. Reactive oxygen species (ROS) was directly synthesized in plants exposed to blue light [76]. The long exposure to blue light might generated excess ROS in the B-treated *C*. *asiatica*. As a result, the expression of flavonoid biosynthetic genes and the production of flavonoid were increased to respond to excessive ROS.

**Fig 5:**
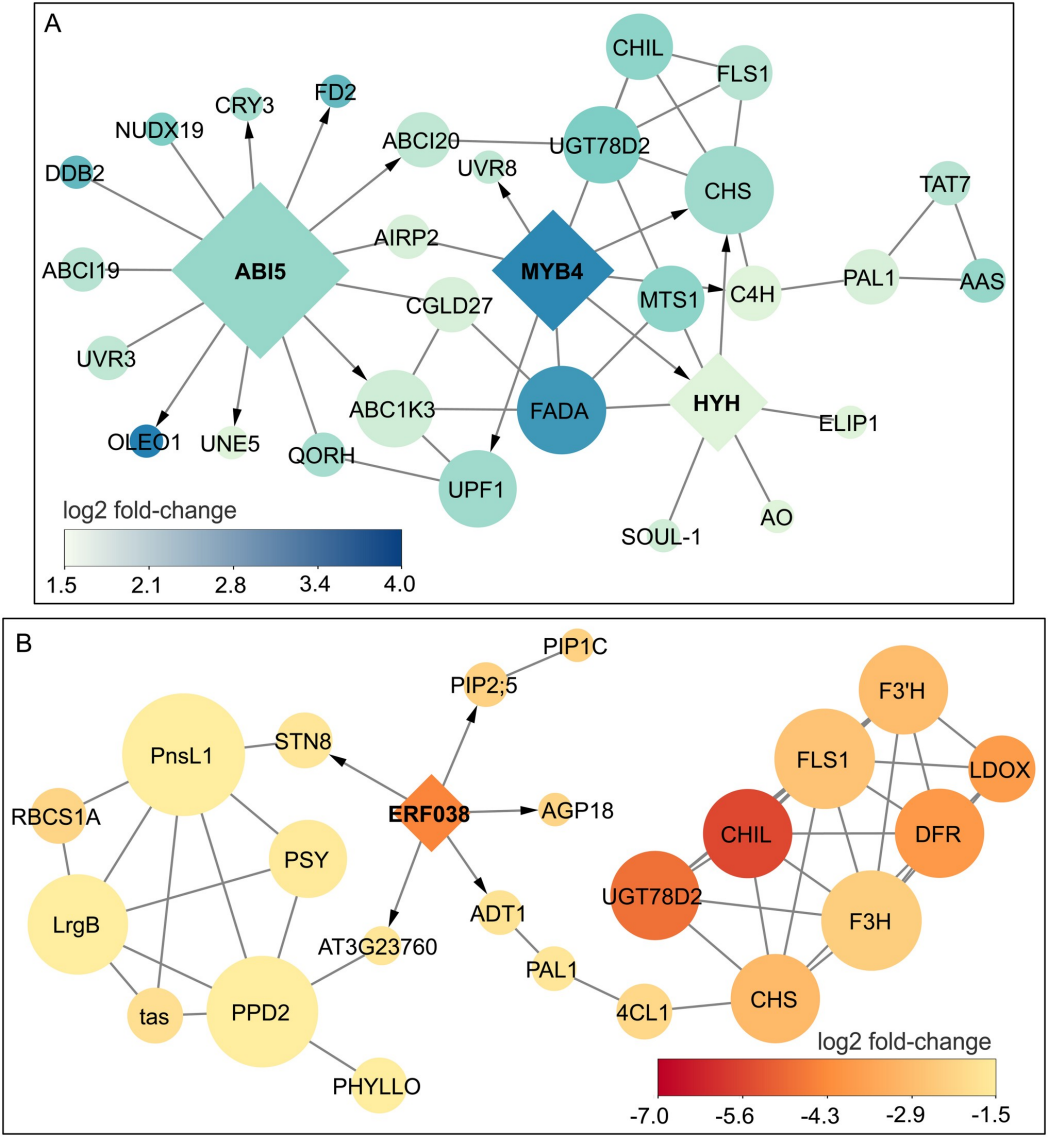
The networks of the DEG-encoded proteins. The network of the proteins encoded by the DEGs (A) under blue and (B) red light are shown. The regulation relationships are shown by directed edges connecting TF nodes (diamond shape) to target gene nodes (circle shape). The co-expression relationships are represented by undirected edges between non-transcription factor nodes. The size of a node is proportional to the number of edges that connect to that node. The full lists of genes are in S4 Table.

To identify transcription factors (TFs) that were involved in the regulation of flavonoid biosynthetic gene expression, we built the networks of all DEG-encoded proteins (Fig 5). We found three TF hubs among the blue-upDEGs, including ABSCISIC ACID-INSENSITIVE 5 (*CaABI5*), Myeloblastosis (MYB) family transcription factor 4 (*CaMYB4*), and *CaHYH* (Fig 5A). *CaABI5* showed connections with *CaCRY3*, *CaUVR3*, and *CaDDB2* (Fig 5A), which had an *ABI5* binding site in their promoter sequences. *CaMYB4* was a R2R3-type MYB protein, which could play a role in the expression of *ABI5* and several genes of the flavonoid biosynthesis pathway [77, 78]. In this study, *CaMYB4* encoding gene was co-upregulated with the cinnamate 4-hydroxylase (*CaC4H*), chalcone synthase (*CaCHS*), Probable chalcone-flavanone isomerase 3 (*CaCHIL*) and other flavonoid biosynthetic genes. *CaHYH* was found as a counterpart of *CaELIP1*, *CaMYB4*, and *CaCHS* in the network. Hormone Abscisic acid (ABA) has been reported to promote the synergy of *ABI5*, *MYB*, *HY5/HYH* in response to salinity stress [79]. In this study, the expression of the β-glucosidase encoding gene (*CaBG*), which was involved in ABA metabolism, was higher under blue light than that under red light. β-glucosidase was suggested to steeply increase local ABA concentrations to initiate early light stress responses, including the biosynthesis of flavonols [39]. In addition, we found a higher expression of the *CaMYB12* encoding gene under blue light than that under red light (Fig 2G). The *MYB12* transcription factor could activate the expression of *CHS*, *CHI*, *F3’H*, and *FLS* [80].

In the network of proteins from red-down DEGs, Ethylene-responsive transcription factor 38 (*CaERF38*) was only one TF hub (Fig 5B). This TF was co-downregulated with State transition 8 (*STN8*), which was essential for PSII repair [61]. *CaERF38* is also connected to Arogenate dehydratase 1 (*CaADT1*), which was involved in phenylalanine production [81]. Phenylalanine is a substrate of PAL1 in the phenylpropanoid biosynthesis pathway (Fig 4A). *CaPAL1* was oppositely expressed in the R-treated and the B-treated *C*. *asiatica* (Fig 2D and S4 Table). Under red light, *CaPAL1* was co-downregulated with 4-coumarate—CoA ligase 1 (*Ca4CL1*), and a cluster of genes involved in the biosynthesis of flavonoids (Fig 5B). The expression of transcription factor *ABI5*, *MYB4*, and *ERF38*, and hormone ABA and ethylene was induced under stress conditions that could cause oxidative stress in plants [79, 82, 83]. The expression of these TFs might contribute to the different expressions of flavonoid biosynthetic genes between the R-treated and the B-treated *C*. *asiatica* to respond to ROS productions that could be higher under blue light [76].

### Expression analysis of selected DEGs by RT-qPCR

We selected ten DEGs, including *CaCRYD*, *CaUVR3*, *CaBIC1*, *CaELIP1*, *CaVDE*, *CaABA4*, *CaPAL1*, *CaCHIL*, *CaF3H*, and *CaABI5*, for RT-qPCR validation analysis. The plot of gene expression fold changes calculated from RT-qPCR data and RNA-seq data showed high correlation coefficient value (R^2^ = 0.98) (S3 Fig). This result indicated that differential expression analysis results from RT-qPCR data and RNA-seq data were highly consistent.

## Conclusion

In this study, we investigated the changes in *C*. *asiatica* gene expression levels in response to monochromatic red (low energy) or blue (high energy) light. A notable difference between *C*. *asiatica* plants under different light conditions was the different expression profiles of stress responsive genes, which were up-regulated under blue light but were down-regulated under red light. Some of these genes were involved in DNA damage repairs (*CaCRYD* and *CaUVR3*), PSII photodamage responses (*CaELP1*), and xanthophyll biosynthesis. The expression levels of several genes in flavonoid biosynthesis pathway were higher under blue light compared to red light. The total flavonoid contents measured were in agreement with the difference in gene expression levels. Increased expression levels of *CaABI5*, *CaMYB4*, *CaMYB12*, and *CaHYH* TFs appeared to correlate with the expression levels of flavonoid biosynthetic genes under blue light, while the down-regulation of *CaERF38* might correlate with reduced expression levels of such genes under red light. The expression levels of several photosynthetic genes were also different between blue light and red light and might be associated with the difference in the flavonoid levels. Overall, our results showed different expression profiles of several high-light induced TF, signaling, and stress-responsive genes under different light conditions. To further elucidate the responses of *C*. *asiatica* to different light treatments, the levels of high-light induced stresses and photosynthetic efficiency should be measured. HPLC runs should be carried out to precisely measure the levels of several intermediates in the flavonoid biosynthesis pathway.

## Supporting information

S1 Fig *C*. *asiatica* plant samples. Representative *C*. *asiatica* plants that were treated with (A) monochromatic red light (compared to that treated with white light) and (B) monochromatic blue light (compared to that treated with white light) for five days are shown.
(TIF)

S2 Fig Overview of the total number of DEGs. (A) The Venn diagram of the up-and the down-regulated DEGs under red and blue lighted is shown. The Venn diagrams of the up-regulated DEGs and the higher expressed DEGs under red (B) and blue light (C) are shown.
(TIF)

S3 Fig RT-qPCR validation. Scatter plot shows gene expression fold changes calculated from RT-qPCR data and RNA-seq data. The correlation coefficient (R^2^) value is = 0.98. The fold changes are from the comparison between the R-treated and the W-treated *C*. *asiatica* (red circle), the B-treated and the W-treated *C*. *asiatica* (blue triangle), and the B-treated and the R-treated *C*. *asiatica* (orange square).
(TIF)

S1 Table Composition of Enshi medium solution. (XLS)

S2 Table RT-qPCR primers. (XLS)

S3 Table RNA sequencing and read mapping statistics. (XLSX)

S4 Table Lists of all differently expressed genes. Differently expressed genes from the comparisons between (A) the R-treated and the W-treated *C*. *asiatica*, (b) the B-treated and the W-treated *C*. *asiatica*, and (C) the B-treated and the R-treated *C*. *asiatica* are shown.
(XLSX)

S5 Table Lists of abbreviations. (XLSX)
