# Supplementary figures and images for: Transcriptome sequencing revealed the influence of blue light on the expression levels of light-stress response genes in Centella asiatica
Source: PLoS One. 2021 Nov 29;16(11):e0260468. doi: 10.1371/journal.pone.0260468 (PMC8629183; doi:10.1371/journal.pone.0260468)

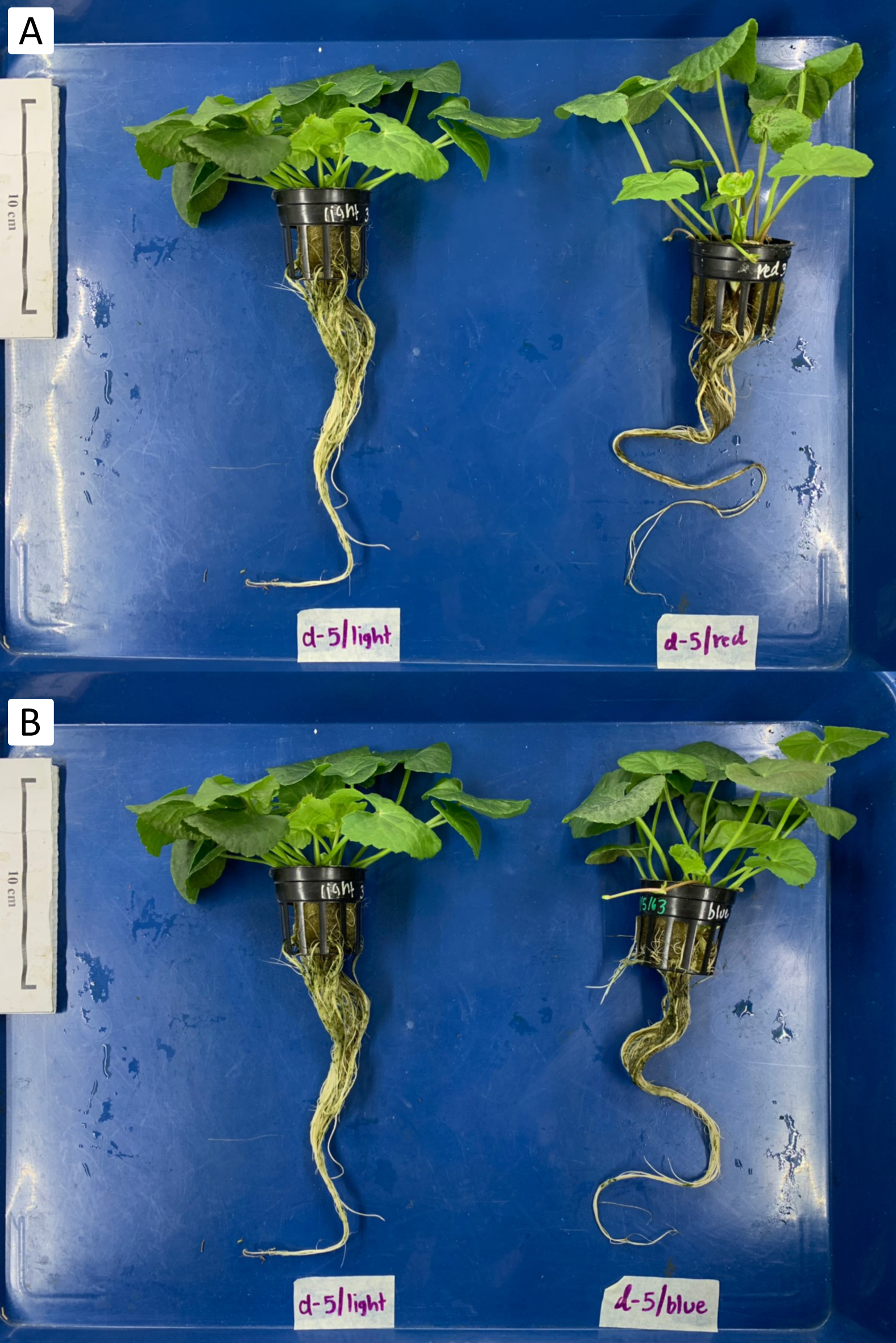

Supplement: S1 Fig — Representative C. asiatica plants that were treated with (A) monochromatic red light (compared to that treated with white light) and (B) monochromatic blue light (compared to that treated with white light) for five days are shown. (TIF) [file pone.0260468.s001.tif]

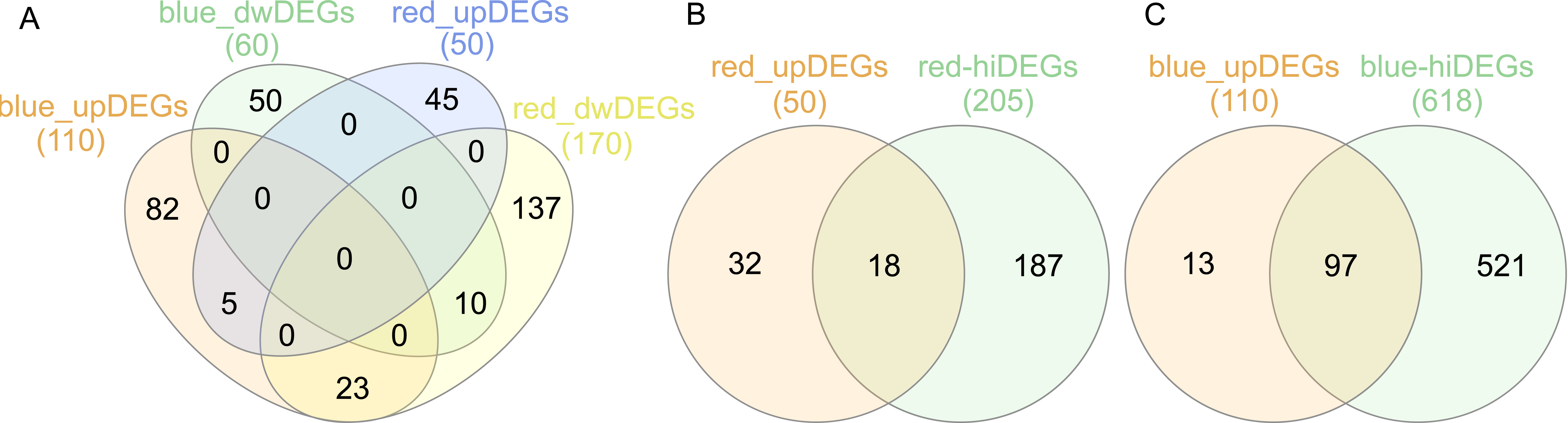

Supplement: S2 Fig — (A) The Venn diagram of the up-and the down-regulated DEGs under red and blue lighted is shown. The Venn diagrams of the up-regulated DEGs and the higher expressed DEGs under red (B) and blue light (C) are shown. (TIF) [file pone.0260468.s002.tif]

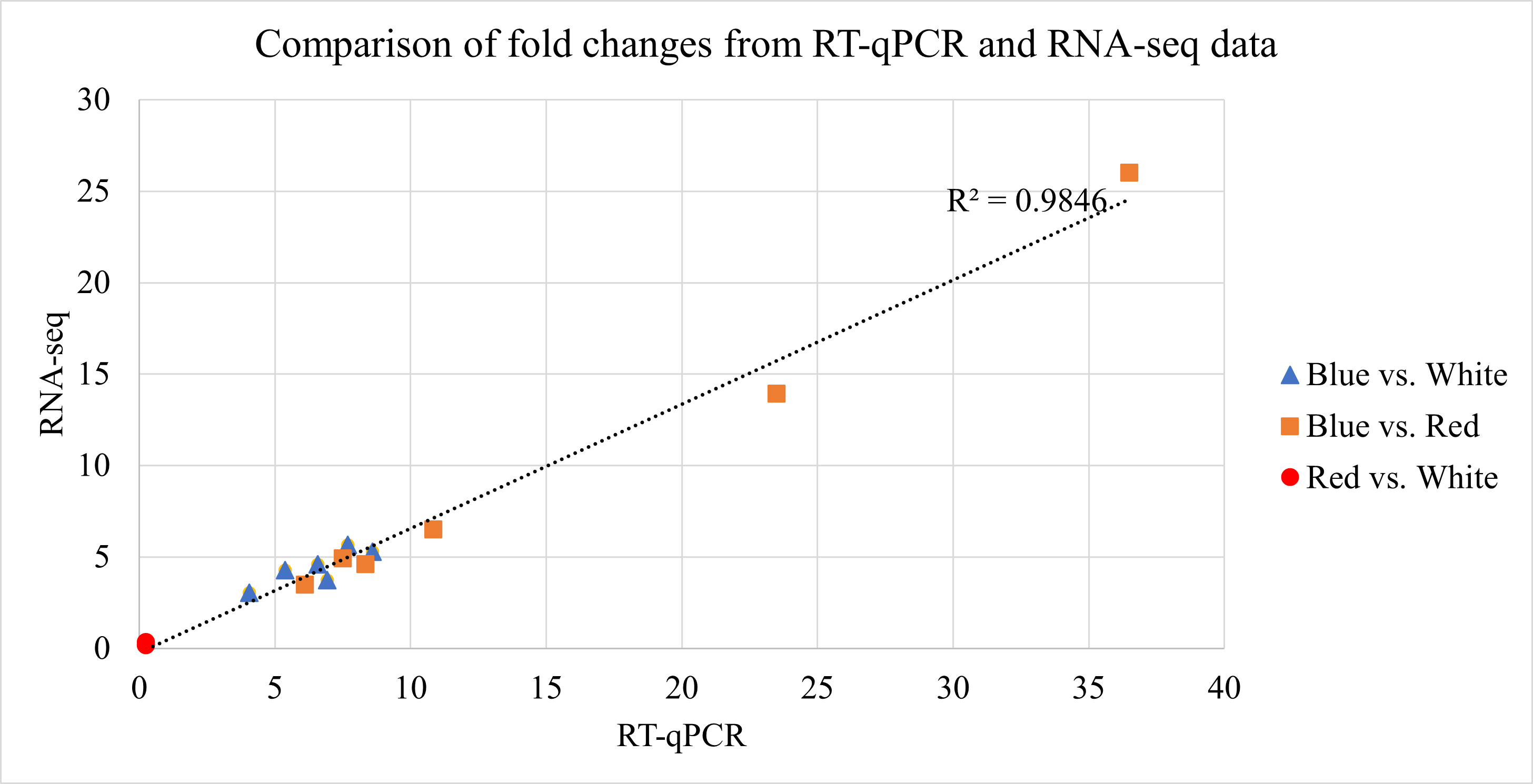

Supplement: S3 Fig — Scatter plot shows gene expression fold changes calculated from RT-qPCR data and RNA-seq data. The correlation coefficient (R2) value is = 0.98. The fold changes are from the comparison between the R-treated and the W-treated C. asiatica (red circle), the B-treated and the W-treated C. asiatica (blue triangle), and the B-treated and the R-treated C. asiatica (orange square). (TIF) [file pone.0260468.s003.tif]
